# Considering the Effects of Cannabinoids and Exercise on the Brain: A Narrative Review

**DOI:** 10.3390/sports13090320

**Published:** 2025-09-11

**Authors:** Amir Yahya Rajaei, J. Patrick Neary, Elizabeth S. Thompson, Jyotpal Singh, Cameron S. Mang

**Affiliations:** 1 Faculty of Kinesiology and Health Studies, University of Regina, 3737 Wascana Parkway, Regina, SK S4S 0A2, Canada; arl550@uregina.ca (A.Y.R.); patrick.neary@uregina.ca (J.P.N.); singhj32@gmail.com (J.S.); 2 College of Pharmacy and Nutrition, University of Saskatchewan, 107 Wiggins Road, Saskatoon, SK S7N 2Z4, Canada; e.thompson@usask.ca

**Keywords:** endocannabinoid, cannabidiol, tetrahydrocannabinol, cannabis, inflammation, vascular, neuroplasticity

## Abstract

Recently, there has been rising interest in the use of cannabis and its derivatives as therapeutic tools to support brain health, particularly in athletes. Cannabis-based substances interact with the endogenous cannabinoid (i.e., endocannabinoid) system, which is involved in widespread physiological processes that contribute to brain function. In other work, the benefits of exercise for brain health have been prominently noted. Despite large bodies of work on both cannabinoid and exercise influences on brain function, there is an understudied overlap in their physiological effects that may be especially important in athletic populations regularly engaged in high volumes of exercise. This narrative review describes mechanistic overlaps between cannabinoid and exercise effects on brain function. The literature search was broad, emphasizing research published since 2010 and including randomized clinical trials, observational studies, case studies, preclinical work, both human and animal studies, and information presented in related review articles. The focal point of the current review is the potentially overlapping effects of cannabinoids and exercise on brain function via physiological processes underpinning inflammation, vascular function, and neuroplasticity. Mechanisms are described in detail with consideration of common and contrasting influences of cannabinoids and exercise on the brain. Altogether, the compiled information suggests that indirect and direct interactions between these two therapeutic avenues have potential to introduce additive, synergistic, or opposing effects. Considering such interactions will be critical in optimizing therapeutic strategies involving cannabinoids as they are increasingly applied in the sport sciences and beyond.

## 1. Introduction

There is increasing public interest in therapeutic applications of cannabis and its derivatives, due in part to its decriminalization in multiple Western countries [1]. Alongside this interest, there is a growing body of research dedicated to enhancing knowledge of the endogenous cannabinoid (i.e., endocannabinoid) system and the use of cannabis-based or related substances as medications and nutritional supplements [2]. Although the endocannabinoid system has widespread involvement in human physiology, its potential to support brain function has drawn particular attention [3,4]. Notable impacts of the endocannabinoid system on brain physiology occur via influence on inflammatory processes [5], vascular responses [6] (pp. 393–422), and neuronal transmission [7]. These effects may have implications for development of strategies to address an array of brain health challenges, ranging from psychiatric to neurological [8,9], including in athlete populations in which there is an urgent need for improved pain management and concussion recovery protocols [10]. Yet many gaps in knowledge remain surrounding the efficacy and long-term effects of therapeutic applications that target the endocannabinoid system, as well as potential interactions with other interventions (e.g., medications, dietary supplements, behavioral strategies).

In other work investigating therapeutic strategies to enhance brain health, research demonstrating the benefits of exercise on brain function is increasingly recognized [11,12,13,14,15]. The physiological effects of exercise are perhaps more wide-ranging than any other intervention. As such, many of the effects of exercise on the brain appear to overlap with those tied to the endocannabinoid system. For example, both habitual and acute exercise are tied to alteration of inflammatory processes [16], vascular responses [17], and neuroplasticity [18], all of which are partly regulated by the endocannabinoid system [19,20]. Moreover, some beneficial effects of exercise on the brain may occur through direct activation of the endocannabinoid system [20,21]. Nevertheless, there has been limited work to date that provides a clear overview of the seemingly vast interactions between the endocannabinoid system and exercise effects on the brain. Understanding such interactions may be particularly relevant to athletes given their regular engagement in high volumes of exercise and increasing exploration of cannabinoid use within sports science [10].

In this narrative review, we aim to improve understanding of the mechanistic overlap of endocannabinoid system activation and exercise effects on brain function. To this end, brain function is broadly defined as the overall operations of the brain, spanning cognitive and non-cognitive activities, and physiological processes. Following a brief description of the endocannabinoid system, focal points of the review will consider overlapping effects of cannabinoids and exercise on inflammation, vascular function, and neuroplasticity. Implications and future directions discussed will emphasize considerations for athletic populations and sports science.

## 2. Literature Search Methods

For this narrative review, our initial literature search focused on articles published between 2010 to present. Recent publications were emphasized to highlight the current state of knowledge in each specific section. However, antecedent sources cited in more recent literature were also considered to understand the extent of recent progress achieved in the field of study. To identify relevant literature, we searched PubMed and Google Scholar with multiple terms. General keywords in these searches included endocannabinoids, cannabinoids, phytocannabinoids, cannabinoid receptors, exercise, physical activity, brain, central nervous system, inflammation, immune system, vascular system, neuroplasticity. Other, more specific keywords included specific cannabinoid types and receptors, acute exercise, regular exercise, long term, short term, immunomodulation, cytokines, neuromodulation, neurotrophins, and neurotrophic growth factors. We combined keywords to consider different contexts and circumstances (i.e., activity of the endocannabinoid system in a specific physiological circumstance). As literature accumulated, we also searched authors whose research focus revolves around the topic of this review. We included randomized clinical trials, observational studies, case studies, and preclinical work, considering both in vitro studies conducted in human and animal cells, and in vivo studies including human and animal research separately. We also considered information presented in prior related reviews. Titles and abstracts of identified articles were screened by two authors (A.Y.R., C.S.M.). Articles were excluded if redundant or if they presented duplicate information. The authors verified the methodological quality of included references by considering the academic journal credibility and the alignment of methods, results, and conclusions. Overall, 243 references related to the endocannabinoid system, exercise, and brain function were included. Across these references, 184 were published between 2010 and 2025. Moreover, 141 focused on the endocannabinoid system (70 original research articles; 71 reviews, book chapters, or public reports), 69 focused on exercise or sport science (27 original research articles; 42 reviews), and 12 considered both the endocannabinoid system and exercise in some respect (9 original research articles; 3 reviews). The remaining 21 articles explored related topics such as inflammation, vascular function, and neuroplasticity. While we acknowledge the potential bias of a narrative review approach, the format and supporting search methods were selected to present a broad, up-to-date perspective on a generally underexplored topic area and provoke thought and future work by communicating major findings, study differences, and gaps in knowledge [22,23].

## 3. Overview of the Endocannabinoid System

Recent advances in understanding the endocannabinoid system have established a foundation from which novel strategies might be developed to exploit its activation for the benefit of various aspects of human health and performance, including brain function. It is a vast cell-signaling system comprising endocannabinoids, their receptors, and the enzymes responsible for their synthesis, activation, and degradation [24]. The endocannabinoid system performs widespread regulatory activities, suggesting a primary role in maintaining homeostasis across the body and brain [25]. Yet, the multifunctional nature of many components of the system are increasingly recognized and critical to consider for development of therapeutic applications.

The term cannabinoid refers to a group of chemical substances that bind cannabinoid receptors, and may be divided into three groups: endogenous cannabinoids, phytocannabinoids, and synthetic cannabinoids [26] (pp. 461–471). Endogenous cannabinoids, or endocannabinoids, are activity-dependent signaling molecules which are produced de novo and have regulatory roles in multiple organs, mainly the central nervous system (CNS) and the immune system [27]. Phytocannabinoids are naturally occurring molecules that are found in the cannabis or hemp plant (*Cannabis sativa*), with Δ^9^-tetrahydrocannabinol (THC) and cannabidiol (CBD) being the most common examples. Synthetic cannabinoids (e.g., Dronabinol, Nabilone) refer to a class of laboratory-manufactured substances that chemically resemble cannabinoids and are produced for medical use or, in some cases, misused recreationally [28,29]. All three groups of cannabinoids may interact with receptors of the endocannabinoid system, of which the family of G-protein-coupled receptors (i.e., metabotropic) are the most well-studied [30] and known as cannabinoid receptors 1 and 2 (CB1 and CB2). Of note, synthetic cannabinoids generally interact most strongly with endocannabinoid receptors, which can lead to discrepancies in downstream effects on physiological function, health, and behavior [31,32].

CB1 may be considered an important target to influence brain function via the endocannabinoid system given that it is primarily found in the CNS. It is prevalent in presynaptic neurons of the cerebral cortex, basal ganglia, dorsal root ganglia, and spinal dorsal horn [25], but is also present in other parts of the body, including the peripheral nervous system, the gastrointestinal tract, and reproductive organs [33]. In contrast to CB1, CB2 is primarily distributed throughout peripheral tissues, such as immune and endothelial cells, as well as in cardiovascular and gastrointestinal organs [34], although it can be found on glial cells and astrocytes in the CNS [35,36] (pp. 42–47). CB2 influences within the immune and vascular systems are indirectly tied to brain function and will be highlighted in subsequent sections. Outside of CB1 and CB2, another family of endocannabinoid receptors are the transient receptor potential cation channel subfamily. The most studied receptor of this type is the transient receptor potential vanilloid receptor type 1 (TRPV1). TRPV1 is abundant in the cardiovascular and peripheral nervous systems as well as cerebrovascular spaces [37].

**Figure 1:**
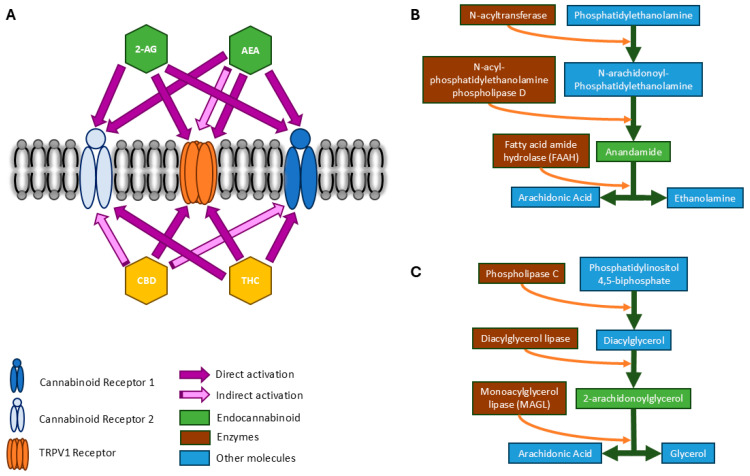
Schematic of major components of the endocannabinoid system. Panel (**A**) depicts the interaction of different cannabinoids and cell receptors. AEA activates CB2 as a primary agonist and is a partial agonist of CB1. High concentration of AEA activates TRPV1 directly and indirectly. 2-AG activates CB1 and CB2 as an agonist for both and activates TRPV1 at a higher concentration than AEA. Phytocannabinoids (described in more detail below) can also activate the endocannabinoid system. CBD indirectly activates CB1 and CB2 by influencing fatty acid amide hydrolase (FAAH) and monoacylglycerol (MAGL), and by binding to the allosteric site. CBD also activates TRPV1 at low concentrations. THC binds directly to CB1, CB2, and TRPV1 as a partial agonist. Panels (**B**,**C**) depict mechanisms of synthesis and degradation of AEA and 2-AG. The on-demand mechanism of endocannabinoid supply involves degradation and synthesis of enzymes. Phosphatidylethanolamine is the precursor of AEA. FAAH is the key enzyme that degrades it. Phosphatidylinositol is the precursor of 2-AG, and MAGL is its degradation enzyme.

The endocannabinoids that interact with these receptors are cellular messengers that are mainly derived from cellular membrane phospholipids. Contrary to many neurotransmitters and neuropeptides, endocannabinoids are not stored in pre-formed synaptic vesicles. Instead, they exist in the form of precursors within cells, while extracellular and intracellular calcium concentration mechanisms, such as elevation of intracellular calcium, triggers ‘on-demand’ synthesis of the endocannabinoids, and subsequently prompts their degradation [5,38]. The rapidity of the on-demand synthesis and degradation sets the endocannabinoid signaling system apart from conventional neurotransmitter and hormonal signaling [24]. Several endocannabinoids have been identified, of which two are known to have major impacts on the CNS and the immune system. These endocannabinoids include N-arachidonoyl ethanolamine (AEA), commonly known as anandamide, and 2-arachidonoylglycerol (2-AG). Both AEA and 2-AG act as retrograde messengers [10,39]. AEA initiates a relatively slow retrograde signal and is a low-affinity selective ligand for CB1 [35,40] (pp. 42–47). 2-AG is the most abundant endocannabinoid in the brain and provides a rapid and temporary retrograde signal. It has shown a similar affinity towards both CB1 and CB2 [38]. While phytocannabinoids and synthetic cannabinoids are administered exogenously to activate CB receptors, AEA and 2-AG synthesis and binding is promoted through endogenous stimuli, such as the physiological effects of exercise [21,41]. Key elements of the endocannabinoid system are depicted in Figure 1, including interactions between cell receptors and cannabinoids (A) and synthesis and degradation of endocannabinoids (B and C).

## 4. Targeting the Endocannabinoid System to Alter Brain Function

The prevalence of endocannabinoids and their receptors throughout the CNS and immune system, and the potential for exogenous activation of the endocannabinoid system via phytocannabinoids, has led to conjecture regarding potential therapeutic applications for brain function. The two most abundant and well-studied phytocannabinoids, THC and CBD, interact with endocannabinoid receptors (CB1 and CB2), and both have shown psychoactive properties, although only THC is considered an intoxicating substance [42]. THC is a partial agonist for the CB receptors, primarily binding to the main orthosteric site of CB1, the extracellular area formed by the transmembrane helices [43], to elicit its psychoactive effects [44]. CBD, on the other hand, appears to interact indirectly through the allosteric binding site of the CB receptors. CBD acts as a non-competitive negative modulator of THC and the effects of 2-AG [45], such that the effects of THC are largely balanced by CBD’s actions on CB1 [10]. Both THC and CBD act as agonists to CB2 [46] and demonstrate affinity towards the TRPV1 endocannabinoid receptors of the cardiovascular and peripheral nervous systems [47]. These interactions are presented visually in Figure 1A.

Notably, evidence suggests that physical exercise can also provide a stimulus to activate the endocannabinoid system without administration of exogenous substances [20]. Indeed, brain function is influenced by inflammation, vascular response, and neuroplasticity, all of which are influenced by exercise and regulated in part via the endocannabinoid system. Inflammation in the CNS can suppress neurogenesis [48,49], disrupt growth factor signaling [12,50,51], and promote neurodegeneration [52], among other deleterious effects. Likewise, vascular function is critical to ensure delivery of oxygen and nutrients and clearing of waste or pathogenic molecules within the CNS [53]. Finally, neuroplasticity refers to the capacity of the CNS to undergo adaptive structural and functional change, a process that is critical for preserving and supporting brain function [54]. In the following subsections, we will consider the influences of the endocannabinoid system on each of these processes, exploring effects of exogenous activation via phytocannabinoids and endogenous activation via exercise, and consequences for brain function.

## 5. Regulating Inflammation

Inflammation is a function of the body that attracts the cells of the immune system from circulation to areas where they are required to eliminate hostile agents, such as pathogens or damaged cells, and thus restore homeostasis [55]. The first cells which are brought about to the site of the inflammation are neutrophiles, followed by monocyte, lymphocytes, and mast cells [56]. Considering brain function, neuroinflammation is a hallmark of many neurological conditions and may accumulate via immune activity in the CNS, autoimmune reactions, or systemic inflammation from the rest of the body. However, the relationship between inflammation and neuroinflammation is complex and influenced by many factors, including diet [57], stress, sleep [58], and physical activity [59]. While regular physical activity is generally anti-inflammatory, the physical stress of intense exercise and sport-related injury introduce inflammation and associated health risks that must be monitored and managed to optimize athletic performance and training adaptations [56,60,61]. Endocannabinoid signaling is highly involved in immunoregulation related to inflammation, including neuroinflammation. From involvement in cell functions like apoptosis and proliferation to influences on cytokine production and release, endocannabinoid signaling is generally associated with downregulation of pro-inflammatory, and upregulation of anti-inflammatory, responses. Nevertheless, the type of cell and the activated receptor ultimately determines the type of inflammatory response that is promoted.

CB2 receptors are more prominent in the immune system than CB1 receptors and are expressed on multiple cells including B-cells (white blood cells), neutrophils, and natural killer cells [5,62]. CB2’s activation typically promotes immune suppression via an increase in anti-inflammatory factors, decrease in pro-inflammatory factors, and an increase in cytokine production and regulation [63,64]. In contrast, CB1 activation bolsters pro-inflammatory activity [20] or anti-inflammatory activation depending on the context. For example, in neuronal injury animal models that are relevant to concussion in humans, CB1 ligands capable of activating the receptor (i.e., agonists) reduce glutamate-mediated excitotoxicity and have anti-inflammatory effects [65].

AEA and 2-AG have also been studied for their specific roles in inflammation. AEA inhibits pro-inflammatory responses by blocking lymphocyte T cells’ migration, and suppressing monocytes and microglia cells from production and release of pro-inflammatory cytokines, such as interleukin-2 (IL-2), interleukin-6 (IL-6), and interferon gamma (IFN-γ) [66]. High concentrations of AEA have also been shown to block macrophage cell migrations [67]. In animal models, 2-AG was shown to counteract inflammatory effects through its influence in the mitogen-activated protein kinase (MAPK) pathway [68,69], blocking of immune cell proliferation [70], formation of antibodies [71], and mobilization of the natural killer cells [70]. Together, the modulatory actions of AEA and 2-AG reduce inflammatory effects of cytokines and immune system activity [72] that may otherwise disrupt brain function.

Several animal studies have indicated that the intake of phytocannabinoids can modulate inflammatory activities via the endocannabinoid system [73]. For example, CBD and its analogs induced significant decreases in IL-1, IL-6, and tumor necrosis factor-α (TNF-α) concentration in animal models [74]. Other research exploring inflammation in animal models of neurological disorders (e.g., Parkinson’s and Huntington’s disease) suggests that co-administration of CBD and THC reduces inflammation and slows disease progression [75]. In the context of sport, studies of CBD administration alongside strenuous exercise training have shown little evidence to date of an anti-inflammatory effect [76,77,78] but the research is scarce and its known effects on cytokines and cortisol are still recognized as potential avenues to mitigate inflammation induced by exercise [79]. Evidence generally suggests that THC alone does not alter pro- or anti-inflammatory cytokines [73]; however, factors such as dose, mode of consumption, and age may need to be considered [80]. In rodent models, chronic intake of THC during adolescence induced an increase in pro-inflammatory factors like TNF-α and a decrease in anti-inflammatory factors like interleukin-10 (IL-10) in the CNS, potentially due to long-term upregulation of CB2 receptors on glial cells and a downregulation of CB1 receptors on other cells [81]. Other study in rats suggested that THC exposure in vivo could lead to a macrophage infiltration into adipose tissue that increases TNF-α expression [82]. Table 1 provides a brief summary of findings related to THC administration and inflammatory responses in murine models.

As alluded to above, inflammation is also largely influenced by exercise. Direct effects of exercise on inflammatory activity include activation of macrophages and neutrophils that repair and build muscle and the secretion of cytokines from muscle (i.e., myokines) that support hypertrophy and myogenesis [87]. The myokines also activate secondary messengers and cascades, including actors in the MAPK pathways, that boost immunity [16]. Generally, the myokines released with exercise are anti-inflammatory, such as IL-10, and inhibit production of pro-inflammatory cytokines (e.g., TNF-α) [87,88]. Further, the myokines secreted with exercise elicit increases in growth factors, such as insulin-like growth factor-1 (IGF-1) and brain-derived neurotrophic factor (BDNF), which counter inflammation and provide neuroprotective effects.

A growing body of work indicates that the endocannabinoid system is likely an important contributor to, or mediator of, the effects of exercise on inflammatory activity, including neuroinflammation. For example, a single bout of treadmill running [41] or cycling [21] increases AEA concentration in systemic blood. With its lipophilic profile, AEA has potential to pass through the blood–brain barrier and induce central effects [19]. Notably, aerobic exercise effects on AEA are optimized at moderate intensities, but attenuated at low and high intensities [41]. In other work, 12 weeks of resistance training amplified the expression and activity of CB1 and CB2 in skeletal muscle in older adults [89], and as such, their potential influences on the inflammatory pathways [20]. Exercise also modulates the expression of CB receptors in immune system cells, such as natural killer cells and lymphocytes [90]. These findings demonstrate extensive interplay between exercise, the endocannabinoid system, and inflammation in potentially supporting brain function.

While inflammation is generally a protective physiological function, there are instances in which it becomes excessive and may contribute to, rather than counteract, pathology [91]. Unlike most other cells, neurons have minimal capacity for repair after damage [92] and disproportionate inflammatory responses, such as in cases of illness or injury, can be of substantial detriment [93]. Excessive inflammation also has the potential to disrupt the blood–brain barrier and permit peripheral inflammatory factors to migrate into the CNS [94,95] where they could disrupt neuronal transmission [96], regulatory growth factor signaling [97], mitochondrial function [98], and ultimately contribute to degeneration of neurons [99]. As such, the robust and generally anti-inflammatory effects of both phytocannabinoid intake and engagement in habitual exercise are notable, especially when considered in combination. For instance, both phytocannabinoids [74] and habitual exercise [88] may suppress production of pro-inflammatory cytokines (e.g., TNF-α) and exercise may enhance anti-inflammatory cytokines (e.g., IL-10) [88], providing an additive effect to mitigate systemic and central inflammation. Likewise, increases in AEA and 2-AG with exercise [21,41] may attenuate immune cell migration and proliferation [72], while exercise-induced increases in CB1 and CB2 expression [89] could provide greater opportunity for endocannabinoid system activation via phytocannabinoid intake. An overview of cannabinoid and exercise influences on the immune system is provided in Table 2. Although not yet explored in combination, these plausibly complementary effects could provide major contributions to regulation of cytokines and other inflammatory immune system activity that influence brain function.

## 6. Regulating Vascular Function

The vascular system uses the heart and blood vessels to transport oxygen-rich blood and nutrients, remove low-oxygen blood and waste products, and circulate immune cells throughout the body and brain. Vascular function influences brain function largely through regulation of cerebral blood flow. Human vascular function is regulated by various physiological pathways, including those that comprise the endocannabinoid system. Documented endocannabinoid system effects on the vascular system range from alteration of blood pressure [6], to influences on heart rate variability [47], to moderation of cerebral blood flow under conditions of hypercapnia and hypoxia [114], each of which may play a role in athletic performance and training adaptation to exercise. Nevertheless, it has been suggested that endocannabinoid effects on vascular function may be most critical under pathologic conditions when its homeostatic and potentially cardioprotective effects become increasingly active [115].

Within the vascular system, CB receptors are primarily expressed in the endothelium and myocardium, where they have varied effects [116]. Generally, activation of CB receptors decrease myocardial contraction, promote vasodilation, reduce blood pressure [47,117], and suppresses release of pro-inflammatory factors [6,118]. While these CB receptor effects may indirectly support brain function, CB receptors may also impact the brain more directly through involvement in cerebrovascular regulation [114]. Endothelial cells, neurons, and glial cells release peptides and hormones that influence blood vessel diameter. Each of these cell types expresses CB receptors and releases endocannabinoids (i.e., AEA and 2-AG) that may modulate cerebral circulation bi-directionally [114]. CB1 activation generally suppresses smooth muscle contractility and promotes vasodilation to increase cerebral blood flow, but under stress (e.g., hypoxia) activation of CB1 receptors on neurons induce a decrease in cerebral blood flow [114]. Other work demonstrates that CB2 receptor activation in coronary endothelial cells may attenuate inflammation and increase blood perfusion in cerebrovascular pathology, such as subarachnoid hemorrhage and traumatic brain injury [119,120]. Related research demonstrates a role for AEA in modulating systemic arterial pressure, heart rate, and cerebral blood flow [121,122]). Thus, as with other aspects of endocannabinoid system function, the type of receptor activated and the condition of activation determines specific vascular effects that regulate cerebral blood flow and contribute to maintenance of a healthy extracellular neural environment.

Studies of cannabinoid intake provide additional evidence for influences on vascular function and cerebral blood flow. Use of cannabis [123] and acute administration of THC [124,125] increased cerebral blood flow in the majority of studies in humans and animal models [126]. Discrepant findings [127,128] (p. 50) have been explained by the diverse actions of the endocannabinoid system, such that a vasodilatory effect of cannabinoids on cerebral vessels may increase cerebral blood flow while a suppression of synaptic activity by cannabinoids may decrease cerebral blood flow demands [114]. While acute THC-predominant cannabis use causes hypertension, tachycardia, and an increased risk of cardiovascular events like stroke and myocardial infarction [20,129], chronic cannabis use is not associated with hypertension and cardiovascular disease [130,131]. Chronic THC use is known to cause a general decrease in heart rate and increase in blood volume while raising the risks of heart arrythmia in the long term [132] and potentially impairing autonomic cardiac function [133]. Such effects in regular cannabis users indicate that a downregulation of CB2 receptors may occur with chronic THC consumption [134,135]. On the other hand, a case series suggested that administration of CBD and a combination of CBD and THC increased blood pressure variability and supported recovery in females experiencing post-concussion syndrome [136], a population for which cerebral blood flow during exercise has been identified as a potential marker of recovery [137].

Exercise is recognized as a major component of treatment strategies and preventive measures against vascular risk factors and disorders [138]. Habitual aerobic exercise improves the structure and the morphology of the cardiac muscles and increases myocardium wall thickness [139]. Likewise, regular resistance and endurance exercise increases blood flow reserve under stress [140,141] and lowers blood pressure in both healthy [142,143] and hypertensive populations [144,145] (pp. 153–167). Exercise increases blood vessel diameter, prevents production and buildup of plaques, and boosts angiogenesis [17]. As acute exercise increases heart rate and cardiac output, cerebral blood flow is increased to meet the metabolic demand of the brain. With habitual exercise, changes in cerebral blood flow become increasingly refined and responsive [146] through improved brain vascularization and blood vessel adaptation [147,148]. Exercise-induced changes to vasculature and cerebral blood flow are linked to production of proteins, such as vascular endothelial growth factor and endothelial nitric oxide synthase, by smooth muscle and endothelial cells [140,149]. Likewise, increases in catecholamines with exercise support heart function, enhance endothelial repair mechanisms, and promote angiogenesis [150]. Together, these vascular adaptations to exercise enhance cerebrovascular endothelial processes to support brain function.

In many ways, the documented effects of the endocannabinoid system and exercise on vasculature are overlapping and interactive (see Table 3 for overview). Improvements in cardiac output, blood flow reserve, and the structure and morphology of vasculature with habitual exercise [139,140,142], such as in athletes, could potentially mitigate negative effects of cannabis use on hypertension, heart rate, and risk of myocardial infarction [20,129], with subsequent influences on brain function. Nevertheless, recent work demonstrated that anaerobic performance was impaired and risk for cardiovascular disease increased in female athlete cannabis users relative to their non-using athlete counterparts [151], suggesting that detrimental effects of regular cannabis use are not abolished by exercise participation. On the other hand, acute intake of CBD in endurance-trained male athletes improved peak oxygen consumption, blood lactate accumulation, and ratings of pleasure during an exercise test, suggesting that CBD supplementation may provide beneficial physiological and psychological responses to exercise [77].

Exercise effects on vascular function that influence the brain are likely mediated, in part, by the endocannabinoid system. For example, exercise-induced increases in systemic levels of cannabinoids [21,41] and CB receptor sensitivity [165] may promote binding to CB receptors in the vascular system, such as on endothelial and smooth muscle cells, to promote vasodilation and related benefits for blood pressure, heart rate, and cerebral blood flow. Likewise, evidence of the importance of the endocannabinoid system in moderating cerebral blood flow under hypoxic and hypercapnic conditions [114] suggests that it also contributes to the control of cerebral blood flow during exercise. Recently, a review by From & Crosby (2025) [166] highlighted the bidirectional relationship between endocannabinoids and nitric oxide in modulating cerebrovascular dynamics. Endocannabinoids influence nitric oxide synthesis and downstream signaling pathways, which regulate vascular tone and cerebral blood flow. Conversely, nitric oxide modulates endocannabinoid levels and their associated signaling cascades, creating a reciprocal interplay [166]. Other work emphasizes exercise-induced increases in nitric oxide synthesis that improve circulation and vascular health [167]. These investigations align with evidence that both exercise pre-conditioning [168] and CB2 activation [119,120] may support recovery from brain injury. While not yet tested, it is then possible that cannabinoid intake and exercise participation could provide complementary vascular effects that may be prophylactic to neurological trauma, such as concussion, and generally supportive of brain function.

## 7. Promoting Neuroplasticity

Neuroplasticity refers to the capacity of the CNS to change and reorganize its connections in response to experience. Changes may include alterations in synaptic efficacy, dendritic branching, and formation of new neurons. Neuroplasticity is critical for brain function, with direct involvement in memory formation and learning, compensation for age-related changes in cognition, and recovery from neurological injury or illness [169]. The endocannabinoid system is highly integrated within the CNS, exhibiting effects on brain function through mechanisms such as depolarization-induced suppression of inhibition (DSI) and depolarization-induced suppression of excitation (DSE). Generally, the primary role of the endocannabinoid system within the CNS is to maintain homeostasis by modulating neuronal activity and synaptic transmission accordingly [170]. Yet behavioral influences of the endocannabinoid system are broad and include effects on memory [171], pain regulation [172], and mood [173].

**Figure 2:**
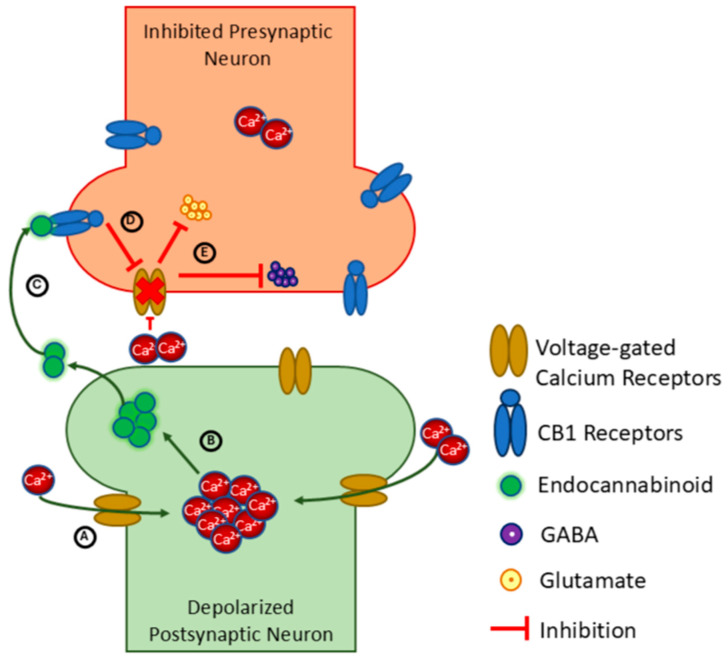
Mechanism of endocannabinoid retrograde signaling and depolarization-induced suppression of inhibition and excitation (DSI and DSE, respectively) in a neuronal synapse. (**A**) Depolarization of the postsynaptic neuron induces calcium ions to enter via voltage-gated channels, increasing the calcium concentration. (**B**) High calcium concentration forces endocannabinoids to enter the intersynaptic space. (**C**) Endocannabinoids bind to CB1 receptors and activate them. (**D**) CB1 activation blocks the calcium channels from opening to calcium ions. (**E**) Membrane potential is not high enough to induce the release of neurotransmitters, hence delaying the GABA-induced inhibition and glutamate-induced excitation.

While all CB receptors can be found in the CNS, CB1 is most abundant in neurons and distributed throughout cortical, subcortical, and spinal regions [25]. CB2 is less prevalent in the CNS, but found in glial cells and astrocytes [35,36] (pp. 42–47), while TRPV1 is located on sensory neurons involved in nociception [174]. DSI and DSE are forms of short-term brain plasticity that depend specifically on CB1 receptor activation [175,176,177,178]. In DSI, as depicted in Figure 2, endogenous cannabinoids are released from the post-synaptic neuron after it is depolarized. The cannabinoids bind to CB1 receptors on nearby pre-synaptic neurons, acting as retrograde messengers to reduce presynaptic release of the inhibitory neurotransmitter gamma-aminobutyric acid (GABA) [175,179,180]. Various studies provide corroborating evidence of cannabinoid-mediated DSI, such as by demonstrating that activation of CB1 through microinjection of AEA inhibited sympathetic nervous system activity [181], and that administration of 2-AG in the rat hippocampus decreased release of GABA [182]. DSE, also depicted in Figure 2, follows a similar mechanism but suppresses the release of the excitatory neurotransmitter glutamate [179] and the postulated involvement of endocannabinoids are less established [176]. Although DSI and DSE were first reported in cerebellar and hippocampal circuits, they have since come to be recognized as relatively universal throughout the brain.

Although DSI and DSE require only a single depolarization to become active and last for only seconds, they may influence mechanisms of long-term neuroplasticity to contribute to more lasting neural change. For example, strengthening of synaptic efficacy by long-term potentiation is facilitated during periods of DSI [183] and endocannabinoids are thought to contribute to induction of long-term depression through DSE [176]. In other work, it was discovered that the N-methyl-D-aspartate (NMDA) receptor, a critical receptor for long-term potentiation in glutamatergic neurons, can combine with CB2 receptors to form a heteromer complex with alternate functionality. In this complex, CB2 receptor activation reduces the signaling output of NMDA receptors, potentially in a manner that combats excitotoxicity, a form of maladaptive neuroplasticity that can be triggered by neuronal injury [184]. Related research demonstrates the neuromodulatory effects of cannabinoids further, with evidence that cannabinoid binding suppresses the release and actions of several neurotransmitters other than GABA and glutamate, including glycine [185], acetylcholine [186], dopamine [187], norepinephrine [188], and serotonin [189]. Yet the conditions of activation must again be considered, as animal studies of drug addiction also suggest that CB1 binding can increase dopamine release in the nucleus accumbens [190].

Consumption of cannabis and its derivatives is widely known to influence neuroplasticity. As noted previously, THC is the primary psychoactive component, with strong affinity to CB1 receptors that are spread across the brain and mainly reduce synaptic transmission [191,192]. Nevertheless, CBD can also be a neuromodulator, such as through the negative allosteric modulation of the CB1 receptor interactions with CB2 receptors, reduced activity of the fatty acid amide hydrolase enzyme, and increases in AEA. Most work in this topic area suggests that regular THC dominant cannabis use disrupts positive, adaptive neuroplasticity. For example, the non-specific activation of CB1 receptors by THC may disrupt the physiological, activity-dependent effects of endocannabinoids and globally suppress release of neurotransmitters that typically promote functional connectivity throughout the brain [193]. On the other hand, human neuroimaging studies suggest that CBD intake may increase both GABA and glutamate concentrations in the basal ganglia and prefrontal cortex [194]. Additionally, studies in mouse models show that CBD intake increases levels of BDNF [195], an important protein involved in neuroprotection and neuroplasticity. Molecular analysis revealed, however, that BDNF increases are dose and location specific. A single CBD dose upregulated BDNF in the medial prefrontal cortex, while repeated dosing increased BDNF in the striatum but slightly decreased it in the medial prefrontal cortex [196]. Given the broad distribution and varied influences of endocannabinoid receptors within the CNS, intake of synthetic or phytocannabinoids has potential to exert a range of effects on neuroplasticity, which depend on factors such as cannabinoid type, dosage, and use pattern, as well as individual characteristics, such as age and sex [192].

A large body of research has also demonstrated a direct impact of exercise on brain function [197,198]. Investigation of gene expression in animal models indicate that nearly 40% of genes upregulated by exercise play a role in mechanisms of neuroplasticity [199,200]. These increases in gene expression enhance production and availability of numerous neurotrophic growth factors (i.e., BDNF) [201] that promote synaptic strengthening, dendritic branching, and neurogenesis [12,200]. Such exercise effects amass to positively alter many characteristics of brain function, such as increased functional connectivity between brain regions [202], increased integrity of white matter [203], and increased brain volume [204,205,206]. Additional research demonstrates that long-term potentiation-like plasticity induced by non-invasive brain stimulation in humans is enhanced in those with high levels of physical activity [207]. The impacts of habitual exercise are underpinned, in part, by the cumulative effects induced by single bouts of exercise. For example, lactate, a byproduct of muscle contraction during acute exercise, may both induce expression of neurotrophic growth factors and be used as fuel within the brain [208,209]. Related studies using transcranial magnetic stimulation indicate that exercise acutely decreases GABAergic and increases glutamatergic activity acutely in the human brain [210,211]. Further studies using non-invasive brain stimulation indicate that various forms of long-term potentiation-like neuroplasticity are increased immediately following exercise in humans [212]. Frequent exposure to these acute, exercise-induced increases in neurotrophic growth factors, decreases in inhibitory activity, and increases in plasticity may then be postulated to evolve into the longer lasting changes in structure and function observed with habitual exercise.

**Figure 3:**
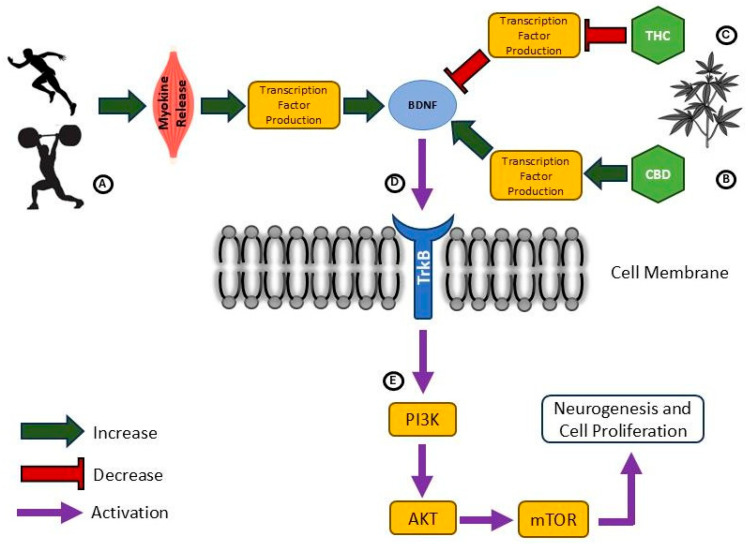
The effects of phytocannabinoids on BDNF expression in neuronal cells. (**A**) Exercise induces the production and release of myokines (e.g., irisin) which then travel through the blood–brain barrier to trigger the production and release of BDNF. (**B**) CBD intake promotes transcription factors that lead to BDNF release. (**C**) THC-induced CB1 activation inhibits the upregulation of factors responsible for BDNF release. (**D**) BDNF binds to the tropomyosin receptor kinase B (TrkB) receptor located on the neurons’ cellular membrane and triggers its activation. (**E**) TrkB receptors turn on the crucial phosphatidylinositol 3-kinase (PI3K) pathway, including protein kinase B (also known as AKT) and mammalian target of rapamycin (mTOR) protein, responsible for growth, proliferation and survival of neurons.

There is increasing evidence for potential interactions between the endocannabinoid system and exercise effects on neuroplasticity. Endocannabinoids that are produced peripherally in response to exercise can cross the blood–brain barrier and exert direct effects on brain function [213], such as those associated with the concept of the “runner’s high” [214]. Likewise, observation of positively correlated increases in peripheral AEA and BDNF immediately and 15 minutes following exercise in male cyclists [215] suggest that activation of the endocannabinoid system might influence expression of neurotrophic growth factors and related effects on neuroplasticity. Figure 3 provides a general overview of influences of phytocannabinoids and exercise on BDNF expression. Considering DSI [175], suppression of GABAergic inhibition by cannabinoids could play a part in reports of cortical ‘disinhibition’ [210,211] and increased long-term potentiation-like plasticity after exercise [216,217] or with high levels of physical activity [207]. On the other hand, evidence for DSE [176] and results demonstrating that THC-dominant cannabis use disrupts long-term potentiation-like plasticity [193] may suggest some alternate effects whereby cannabinoid intake and exercise effects are counter to each other. Further, exercise literature generally demonstrates increases in production and activity of neurotransmitters [200], while cannabinoid binding is more closely associated with a suppression of neurotransmitter release [193]. Nevertheless, these down-regulating effects of cannabinoids are mostly tied to CB1 activation by THC [193] whereas CBD intake largely augments neurotransmitter release [194], such as serotonin (5HT) by way of agonism at the 5HT1A receptor. As such, the complexity of the endocannabinoid system introduces challenges in interpreting how exercise and cannabinoid intake may ultimately interact to influence neuroplasticity. Certainly, exercise supports a neural environment that is conducive to adaptive neuroplasticity [200], including those processes mediated by the endocannabinoid system and triggered by exercise. In Table 4, a summary of cannabinoid and exercise influences within the nervous system is presented. Pending more direct study of cannabinoid-exercise interactions, it may be speculated that the effects of exercise and CBD are somewhat aligned and additive, whereas potentially negative effects linked to regular THC intake may be attenuated through neurophysiological benefits achieved through habitual exercise.

## 8. Implications and Future Directions

Pain management is an area of application of endocannabinoid system study that is pertinent to the sport sciences. Athletes regularly participate in exercise and sporting activities that challenge their physical limits. With these athletic endeavors and associated injuries, they commonly experience pain that may interfere with training, performance, health, and well-being [231,232]. Improved characterization of the role of cannabinoid receptors in nociception, commonly known as pain sensation, has sparked work towards development of pain management strategies that target the endocannabinoid system [233]. For example, CB1 and CB2 receptors are now known to be present in pain centers of the brain, such as the periaqueductal gray of the midbrain [234,235], and the endocannabinoid system has been shown to modulate pain at peripheral, spinal, and supraspinal levels [233]. In a retrospective study of chronic pain treatment with cannabinoids, the use of an oral mucosal spray of THC and CBD (nabiximols) reduced pain intensity among individuals experiencing low back pain, shoulder and neck pain, and failed back surgery syndrome while concurrently decreasing anxiety and stress associated with being in chronic pain [236]. Such analgesic and anxiolytic effects of cannabinoids are thought to be mediated through influences on inflammatory pathways, neurotransmitter release, and neuronal excitability [237].

Related research considers the administration of cannabinoids for prevention and treatment of sport-related concussion [10,238]. Concussion symptoms commonly include dizziness, headache, disorientation and confusion. While symptoms often resolve in 10–14 days, up to 30% of individuals experience persistent post-concussion symptoms lasting months or even years [239]. Physiologically, concussion initiates an excitotoxic, neurometabolic cascade that can impair cerebral blood flow, disrupt autonomic function, and lead to neuronal damage and death [240]. The homeostatic influences of the endocannabinoid system may then exert neuroprotective properties that limit excitotoxicity and inflammation, and promote expression of neurotrophic growth factors (e.g., BDNF) that support recovery [238]. Although such applications are largely unstudied to date, a case report suggested that cannabinoid intake supported recovery from post-concussion syndrome [136].

Alongside research and speculation about such cannabinoid-based applications, exercise strategies have also been developed for management of pain and sport-related concussion. For example, exercise protocols have been demonstrated to be effective for management of chronic low back pain, fibromyalgia, osteoarthritis, and neuropathic pain [241]. Similarly, evidence suggests that symptom-limited exercise protocols may support recovery from concussion and reduce the incidence of persistent symptoms [242,243,244]. These positive exercise effects are thought to relate to activation of endogenous pain modulatory systems, such as the endocannabinoid system, as well as anti-inflammatory, vascular, and neurotrophic effects described in prior sections.

Given that many of the molecular pathways underpinning the effects of cannabinoid intake and exercise participation on brain function are overlapping, it is logical to postulate that the effects of administering cannabinoids are influenced by exercise participation, and vice versa. For example, considerations related to effective dosages of phytocannabinoids, expected availability of receptors and binding of cannabinoids, and ceiling effects of combined strategies may all hinge to some extent on how activation of the endocannabinoid system is concurrently influenced by cannabinoid intake and exercise. Although speculative, it is plausible that habitual exercise could be particularly beneficial in providing an endogenous stimulus that provides guidance for effective physiological use of cannabinoids administered exogenously. Yet, to the best of our knowledge, there is limited empirical research exploring these interactions in either animals or humans. Given efforts to develop therapeutic interventions that target the endocannabinoid system in athletes, future study of these interactions may support efficacy and safety of such strategies.

## 9. Conclusions

The endocannabinoid system is a vast and complex biological regulatory system. Its effects are dependent on many factors, including the tissues, receptors, and cannabinoids involved, as well as the conditions of activation and inter-individual physiology. Endocannabinoid receptors exert a major influence on brain function, both indirectly, through alteration of inflammation and vascular function, and directly, through influences on neuroplasticity. Generally, the homeostatic effects of the endocannabinoid system reduce inflammation and promote measured regulation of cerebral blood flow towards maintaining and supporting a healthy neural environment under both normal and pathological circumstances. In the CNS, the activity-dependent, regulatory effects of the endocannabinoid system may balance adaptive (e.g., strengthening of synapses to support memory) and maladaptive (e.g., excitotoxicity after neurological injury) processes of neuroplasticity. With ‘on-demand’ synthesis of endocannabinoids such as AEA and 2-AG, the endocannabinoid system is continuously active and contributing to various physiological functions. Intake of phytocannabinoids, THC and CBD, provide an exogenous means to further activate the system and potentially accentuate its effects.

Exercise is a powerful intervention to alter brain function through the activation of numerous physiological pathways, including those involved in the endocannabinoid system. Exercise-induced increases in endocannabinoid production and binding [21,41,89] partly underpin the wide-reaching health benefits of exercise for reducing systemic inflammation and improving vascular function that indirectly support brain function. Involvement of the endocannabinoid system in exercise effects on brain function are perhaps most emphatically demonstrated in research examining the “runner’s high” [214]. Although largely unstudied, the endocannabinoid system could plausibly contribute to other known exercise effects on the brain, such as cortical disinhibition and altered expression of neurotrophic growth factors and neurotransmitters.

Both cannabinoid intake and exercise provide means to increase activation of the endocannabinoid system and potentially realize benefits for brain function. Given the homeostatic effects and finely tuned nature of endocannabinoid system signaling, there is some potential that intake of exogenous cannabinoids (i.e., THC) might be disruptive to these processes [200]. Detrimental effects of THC may be mitigated to some extent by CBD and exercise, although CBD effects in particular remain under investigation and may vary by context. Similarly, THC detriments may be mitigated by activation of the endocannabinoid system via exercise. Future research considering interactions between cannabinoid use and exercise will be critical as parallel work exploring the potential benefits of each of these tools for brain function continues to garner scientific, clinical, and public interest.

## Figures and Tables

**Table 1:** Exemplary studies of THC administration and inflammation in murine models.

Model	Dose Intake	Tissue	Outcome	Source
Adult Mice	1–20 mg/kg, twice daily	Cerebellar microglia	Dysregulation of CB1 and microglia activation	[83]
Adolescent Rats	2.5–10 mg/kg, twice daily	Prefrontal cortex	Increase in TNF-α and IL-10 levels	[81]
Adult Rats	10 mg/kg injected intraperitoneally	Adipose tissue	Increase in macrophage infiltration, TNF-α levels and adipocyte hypertrophy	[82]
Adult Rats	Separate 5, 10 and 20 mg/kg daily	Colon (colitis condition)	Decrease in TNF-α, IL-8 and neutrophil infiltration	[84]
Adult Mice	30 µg in 20 µL of acetone applied topically	Ear tissue	Inhibition of interferon γ production, decrease in macrophage migration	[85]
Adjuvant-induced arthritic Rats	2.5 mg/kg daily mixed in sesame oil	Hind paws	Reduction in the concentration levels of TNF-α, IL-1β, and IL-6.	[86]

**Table 2:** Overview of cannabinoid and exercise influences within the immune system.

Immune System Cell	Role(s)	Cannabinoid Influence	Exercise Influence	Source(s)
Lymphocyte B	-Produce antibodies -Keep “copy” of alien antigens	-CBD and THC mediate its inhibition -CB1 activation amplifies antibody production -CB2 activation reduces antibody production	-Acute exercise increases proliferation -Increase production of immunoglobulin	[100,101,102,103]
Macrophage	-Phagocytosis of aliens -Antigen detection	-CBD increases TNF-α secretion -THC reduces release of TNF-α and increases release of IL-1	-Acute exercise is pro-inflammatory -Habitual exercise reduces overall inflammation -Released cytokines modulate macrophage activity	[104,105,106,107]
Neutrophil	-First responders -Phagocytosis of aliens -Tissue repair	-CB1 activation modulates chemotaxis -CB2 activation, CBD, and THC inhibit recruitment to sites of inflammation -THC reduces release of TNF-α and IL-6	-Increased proliferation and mobilization	[108,109,110]
Natural Killer	-Kill harmful cells -Produce and release cytokines	-AEA and 2-AG induce migration and increase cytotoxicity -CBD inhibits its growth -THC reduces killing abilities	-Increased cytotoxicity -Increased proliferation and mobilization -Excessive exercise reduces its impact	[111,112,113]

**Table 3:** Overview of cannabinoid and exercise influences within the vascular system.

Vascular System Cell	Role(s)	Cannabinoid Influence	Exercise Influence	Sources
Cardiomyocyte	-Generate contraction forces of heart -Secrete regulatory proteins (cardiokines)	-CBD prevents heart fibrosis and hypertrophy -CBD prevents Ca^2+^ overload to help with contraction -THC-activated CB1 increase contraction rate and blood pressure. -CB2 promotes survival	-Aerobic exercise increases mitochondrial activity and contraction rate -Promotes regeneration and growth of cardiomyocytes	[132,152,153,154]
Endothelial	-Vascular permeability -Release vasoconstriction and vasodilation factors -Release inflammatory modulators -Release thrombosis factors -Release insulin and lipid metabolism factors -Release vasculogenic and angiogenic factors	-CB2 activation attenuates inflammatory factors -2-AG inhibits endothelium repair and increases inflammation -AEA increases vasodilation -THC impairs endothelial functions including decrease in arterial flow-mediated dilation, and in VEGF-stimulated nitric oxide levels -CBD promotes vasorelaxation, and inhibits angiogenesis, and release of pro-inflammatory factors -THC-activated CB1 promotes release of pro-inflammatory factors, increases influx of Ca^2+^	-Increase nitric oxide production which is responsible for vasodilation and vasorelaxation -Continuous aerobic exercise of various intensities optimizes flow-mediated dilation -Supports repair of tears and wall integrity -Lower circulation of inflammatory factors	[153,155,156,157,158,159]
Pericyte	-Modulate blood flow by modifying capillary diameter and blood–brain barrier passage -Support angiogenesis, myogenesis, and neurogenesis -Exterior protection of endothelium	-Influence blood flow and capillary diameters, especially during ischemic conditions -CBD lowers cellular damage and inhibits release of inflammatory factors in O_2_/glucose deprivation conditions -CB1 affects the vessel diameter and blood flow	-Increase growth rate leading to gains in skeletal muscle mass -Promotes muscle fiber regeneration and angiogenesis -Aerobic exercise enhances contraction force and oxygenation in blood flow	[14,17,160,161]
Vascular smooth muscle	-Regulating blood flow and pressure -Modulating dilation and contraction rates -Maintaining the overall structure by repairing tears and regulating thickness	-CBD inhibits muscle cell proliferation, reduces inflammatory responses and could modulate vasodilation -CB1-mediated THC decreases Ca^2+^ influx, increase vessel diameter	-Aerobic exercise improves contractility and vascular force -Intense exercise strengthens the vascularization wall	[17,162,163,164]

**Table 4:** Overview of cannabinoid and exercise influences within the nervous system.

Nervous System Cell	Role(s)	Cannabinoid Influence	Exercise Influence	Source(s)
Sensory neuron	-Detect internal and external stimuli and transmit signals to CNS	-CB2 activation can regulate neuroinflammation -THC-activated CB1 inhibits neurotransmitter release	-Promotion of growth factor release -Enhanced neurogenesis and overall activity	[193,218,219]
Motor neuron	-Transmit signals from CNS to muscles and glands -Crucial in voluntary and involuntary movements	-Regulate excitatory and inhibitory synaptic transmissions	-Enhanced neuromuscular transmission -Promotion of protein production (e.g., myokines) essential for nerve maintenance	[220,221]
Astrocyte	-Regulate blood and ion flow -Neuroprotection and repair -Synaptic transmission and plasticity	-Activation of CB1 in astrocytes lead to the release gliotransmitters responsible for synaptic functions, and promotion of inflammatory factors	-May change morphology of the cells -Modulate inflammatory response and neuroplasticity	[222,223]
Oligodendrocyte	-Production and maintenance of myelin -Supporting role for nerves by supplying energy -Involvement in immunity	-CB1 and CB2 activation modulates cell differentiation -CBD promotes neuroprotection -THC enhances the myelination process	-Promotes growth factor production -Promotes cell proliferation -Promotes myelin synthesis and repair	[224,225,226,227]
Microglia	-Modulate immune response, remove debris, pathogens -Neurogenesis functions	-CB2 activation is anti-inflammatory -CBD tends to inhibit pro-inflammatory response	-Reduce inflammation -Promote microglia neurogenesis	[228,229,230]
